# Penazaphilones J–L, Three New Hydrophilic Azaphilone Pigments from *Penicillium sclerotiorum cib-411* and Their Anti-Inflammatory Activity

**DOI:** 10.3390/molecules28073146

**Published:** 2023-03-31

**Authors:** Xia Zhang, Yeye Hu, Tao Yang, Xueqing Qian, Weicheng Hu, Guoyou Li

**Affiliations:** 1 Key Laboratory of Environmental and Applied Microbiology, Environmental Microbiology Key Laboratory of Sichuan Province, Chengdu Institute of Biology, Chinese Academy of Sciences, Chengdu 610041, China; 2 Institute of Translational Medicine, School of Medicine, Yangzhou University, Yangzhou 225009, China

**Keywords:** *Penicillium sclerotiorum*, azaphilone, anti-inflammation, fungal pigment, NF-κB signaling pathway

## Abstract

Penazaphilones J–L (**1**–**3**), three new hydrophilic azaphilone pigments, as well as six known compounds, were discovered from the filamentous fungus *Penicillium sclerotiorum* cib-411. Compounds **1**–**3** were structurally elucidated by the detailed interpretation of their 1D and 2D NMR spectroscopic data. Compound **1** is an unprecedented hybrid of an azaphilone and a glycerophosphate choline. Compounds **2** and **3** each contain an intact amino acid moiety. The bioassay showed that compound **3** exhibited significant anti-inflammatory activity. Concretely, compound **3** significantly suppressed the NO production, the expression levels of COX-2, IL-6, IL-1β, and iNOS mRNA in LPS-stimulated RAW264.7 cells. Moreover, treatment of compound **3** prevented the translocation of NF-κB through inhibiting the phosphorylation of PI3K, PDK1, Akt, and GSK-3β. Thus, the inhibition of compound **3** against LPS-induced inflammation should rely on its inactivation on NF-κB.

## 1. Introduction

Fungal metabolites have been an important source for pharmaceuticals and drug lead compounds, such as penicillin (antibacterial), echinocandin B (antifungal), lovastatin (lowering cholesterol), and cyclosporin A (immunosuppressive) [1,2]. Pigments are a large group of secondary metabolites widely found in fungi. Until now, a variety of fungal pigments have been reported, such as carotenoids, melanins, flavins, phenazines, quinones, monascins, violacein, and indigo [3,4], and many fungal pigments such as Arpink Red™ (*Penicillium oxalicum*), riboflavin (*Ashbya gossypii*), and *β*-carotene (*Blakeslea trispora*) have been used in food for decades [3]. Some natural pigments also exhibit excellent biological activities, such as cytotoxicity [5,6], anti-cancer [7], antibacterial activity [8], anti-inflammation [9,10], antioxidation [11,12], and so on. Azaphilones are a typical class of fungal polyketide pigments, featuring a highly oxygenated pyranoquinone bicyclic core, which display diverse bioactivities [13]. Only a small portion of azaphilones contains an isoquinoline core due to the exchange of pyran oxygen by nitrogen [13]. As fungal pigments, azaphilones show promise in the food industry as colorants [14]. 

Our previous study on *Penicillium sclerotiorum* cib-411 discovered a series of new azaphilone alkaloids bearing an *S* configuration at C-7, which displayed significant anti-inflammatory activity [13]. In this study, we aimed at the exploitation of minor azaphilone pigments with high hydrophilicity from this fungus and at investigation of the underlying mechanisms of its anti-inflammation. As a result, three new hydrophilic pigments, penazaphilones J–L (**1**–**3**), together with six known compounds, were obtained from the methanol extract of the fermented rice culture of *P. sclerotiorum* cib-411. Penazaphilone J (**1**) represents a novel azaphilone alkaloid that contains a glycerophosphate choline moiety. Compounds **2** and **3** each contain an intact amino acid moiety. To our knowledge, penazaphilone J (**1**) is the first azaphilone metabolite integrated with a glycerophosphate choline moiety. The isolation and structure elucidation of **1**–**3** and the anti-inflammatory activity of **3** were described here.

## 2. Results and Discussion

### 2.1. Structural Identification of Compounds ***1***–***3***

**Figure 1:**
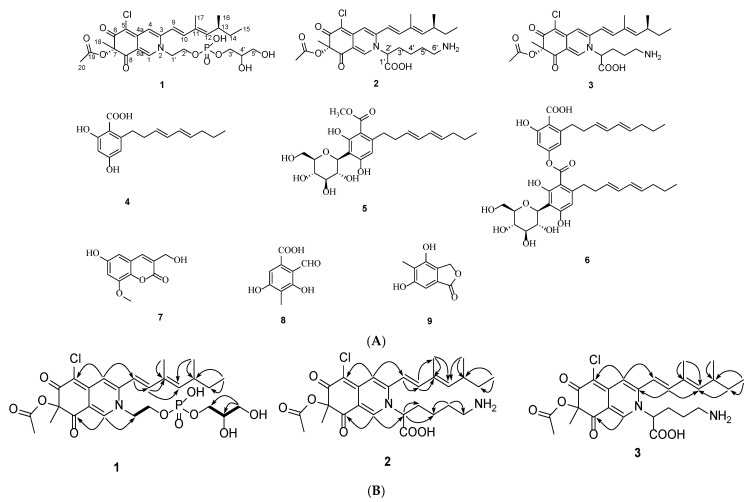
(**A**) The chemical structures of compounds **1**–**9**. (**B**) Key HMBC (arrows) and ^1^H-^1^H COSY (bold lines) correlations of **1**–**3**.

Compound **1** was obtained as a red amorphous powder. The *quasi*-molecular ion peak at *m*/*z* 588.1759 [M + H]^+^ in the HR-ESI-MS indicated a molecular formula of C_26_H_35_ClNO_10_P for **1** with 11 unsaturation degrees. The UV spectrum showed maximal absorptions at 474, 371, and 224 nm. A phosphorus nucleus signal (*δ*_P_ 0.62) was observed in ^31^P NMR, which supported the molecular formula in turn. The ^1^H NMR of **1** showed some diagnostic proton signals for five olefinic methines [*δ*_H_ 8.24 (1H, s), 7.18 (1H, s), 7.11 (1H, d, *J* = 15.4 Hz), 6.58 (1H, d, *J* = 15.5 Hz), and 5.78 (1H, d, *J* = 9.7 Hz)] and five methyls [*δ*_H_ 2.12 (3H, s), 1.94 (3H, s), 1.52 (3H, s), 1.03 (3H, d, *J* = 6.6 Hz), and 0.90 (3H, t, *J* = 7.4 Hz)] (Table 1). Combining with the HSQC and DEPT135 experiments, the carbon resonances in ^13^C NMR spectrum could be differentiated into three carbonyls [two conjugated ketonic carbonyls (*δ*_C_ 194.9, 185.6), and one ester carbonyl (*δ*_C_ 171.6)]; ten olefinic carbon atoms (*δ*_C_ 151.9, 148.8, 148.3, 146.8, 144.6, 134.0, 117.1, 116.4, 112.6, 101.3); one oxygenated quaternary carbon atom (*δ*_C_ 86.2); five methylenes (*δ*_C_ 67.8, 64.5, 63.7, 55.6, 31.1); two methines (*δ*_C_ 72.5, 36.2); and five methyls (*δ*_C_ 23.8, 20.6, 20.2, 12.8, 12.4) (Table 1). The above spectroscopic evidence suggested that compound **1** should be a sclerotiorin derivative [15]. In the HMBC spectrum, H-1 correlated to C-1′, C-3, C-4a, C-8; C-8a, H-4 correlated to C-4a, C-5, and C-9; and H-18 correlated to C-6, C-7, and C-8, which confirmed substructure of pyranoquinone bicylic core. The HMBC correlations of H-9 with C-4, C-10, and C-11; H-17 with C-11 and C-12; H-15 with C-13 and C-14; and H-16 with C-12, C-13, and C-14 suggested the presence of a side chain [C-9/C-10/C-11(C-17)/C-12/C-13(C-16)/C-14/C-15] at C-3. Besides the 21 ^13^C NMR signals accountable for the sclerotiorin skeleton, there remain four methylenes (*δ*_C_ 67.8, 64.5, 63.7, 55.6) and one methine (*δ*_C_ 72.5). Furthermore, the coupling between carbon nuclei [C-1′ (*δ*_C_ 55.6), C-2′ (*δ*_C_ 64.5), C-3′ (*δ*_C_ 67.8), C-4′ (*δ*_C_ 72.5)] and a phosphorus nucleus suggested the presence of a glycerophosphate moiety [16], which was supported by the ^1^H-^1^H COSY cross-peaks of H-1′ with H-2′, and of H-4′ with H-3′/H-5′. Additionally, the HMBC correlation of H-1 with C-1′ suggested that the chain was attached on *N*-2 (Figure 1B).

In the circular dichroism (CD) spectrum of **1**, the negative cotton effect at 384 nm indicated an *S* configuration for C-7 [17]. The stereochemistry of C-13 in **1** was assigned as an *S* configuration from a biogenetic point of view [17]. The double bond between C-11 and C-12 was assigned as an *E* configuration by comparing their NMR values with the known compounds [17]. Compound **1** did not crystallize for the single crystal X-ray analysis. Therefore, the absolute configuration of C-4′ was not determined. Taken together, the structure of **1** was elucidated and given the name of penazaphilone J (Figure 1A). 

Compound **2** was assigned a molecular formula C_27_H_35_ClN_2_O_6_ from the HR-ESI-MS ion peak at *m*/*z* 519.2256 [M + H]^+^. The ^1^H and ^13^C NMR spectra of compound **2** were similar to those of penazaphilone A, but **2** had one more N atom. Careful comparison revealed that compound **2** contained three more methylenes at *δ*_C_ 23.0, 30.6and 55.4 in its ^13^C NMR spectrum, but two doublet methyls at *δ*_H_ 0.92 and *δ*_H_ 0.88 in the ^1^H NMR of penazaphilone A were absent in **2**, suggesting that the leucine was replaced by a lysine. Detailed examination of the HMBC spectrum revealed the key correlations of H-1 (*δ*_H_ 8.19) with C-2′ (*δ*_C_ 55.2); H-2′ (*δ*_H_ 3.67) with C-3′ (*δ*_C_ 31.6) and C-4′ (*δ*_C_ 23.0); and H-5′ (*δ*_H_ 1.83) with C-4′ (*δ*_C_ 23.0) and C-6′ (*δ*_C_ 55.4). confirming the presence of the lysine residue [C-2′(C-1′)/C-3′/C-4′/C-5′/C-6′(NH_2_)] at *N*-2 (Figure 1B). The CD spectrum of **2** also confirmed the stereochemistry of C-7 to be an *S* configuration [(−) *Δε* at 380 nm]. In addition, the configuration at C-13 was biogenetically determined to be an *S* as **1**. Finally, the structure of **2** was established (penazaphilone K) (Figure 1A).

Compound **3** was also purified as a red amorphous solid and had a molecular formula C_26_H_33_ClN_2_O_6_ by HR-ESI-MS analysis (*m*/*z* 505.2079 [M + H]^+^) with 11 degrees of unsaturation. The UV spectrum showed similar maximal absorptions at 479, 372, and 227.5 nm to that of **2**. The ^1^H and ^13^C NMR spectra of **3** also showed similarities to those of **2**. The comparison of the NMR spectra of **3** and **2** indicated that **3** contained one less methene and that the side chain was an ornithine residue [C-2′(C-1′)/C-3′/C-4′/C-5′(NH_2_)]. Detailed analyses of the HSQC and HMBC correlations supported the above speculation. The configurations of C-7 and C-13 were biogenetically determined to be *S* as in **1** and **2**. Therefore, the structure of **3** was elucidated and named penazaphilone L (Figure 1A). 

Penazaphilones J–L belong to sclerotioramine pigments, which contain an isoquinoline skeleton, possibly formed via sclerotiorin, respectively reacting with a glycerophosphate choline, lysine, and ornithine. Fungal secondary metabolites with a glycerophosphate choline moiety have seldom been reported [16]. Penazaphilone J is the first azaphilone pigment that contains a glycerophosphate choline moiety. This study enriches the structural diversity of this class of azaphilones.

The known compounds were identified as 2,4-dihydroxy-6-((3*E*,5*E*)-nona-3,5-dien-1-yl)-benzoic acid (**4**) [18], carnemycin B (**5**) [18], stromecycin (**6**) [18], pestalasin E (**7**) [19], acid II (**8**) [20], and 4-*O*-demethylsilvaticol (**9**) [21] by comparing their spectroscopic data with those reported in the literature.

### 2.2. Effects of Compound ***3*** on the Viability of RAW264.7 Cells

Macrophages are an efficient immune weapon for preventing invading pathogens through phagocytosis or secreting multiple pro-inflammatory cytokines [22,23]. Upon stimulation with LPS, excessive levels of the inflammatory mediator NO can motivate the release of proinflammatory cytokines such as PGE_2_, TNF-α, and IL-6 [24,25]. Therefore, inhibiting the overproduction of these pro-inflammatory cytokines could be a highly effective way to explore new anti-inflammatory agents in vitro.

**Figure 2:**
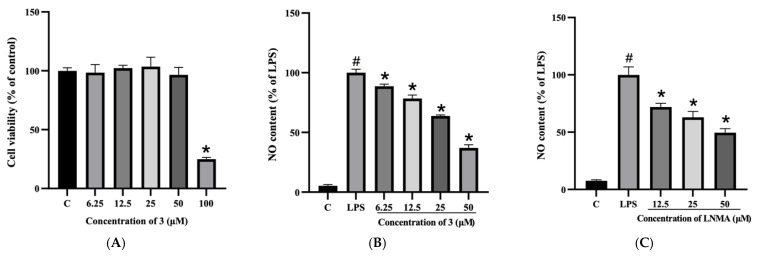
Effect of compound **3** on cell viability and NO production in RAW264.7 cells. (**A**) Cells were treated with different concentrations of compound **3** for 24 h. The cell viability was estimated by MTT assay. (**B**) Cells were pretreated with different concentrations of compound **3** for 30 min and then stimulated with LPS for 24 h. Levels of NO were determined by Griess assay. (**C**) L-NMA was used as positive control and treated as the same. Each value is presented as means ± SD (n = 3). * indicates a significant difference between the LPS group and the drug groups (*p* < 0.05). # indicates a difference between the LPS group and the control group (*p* < 0.05).

Our previous studies showed that penazaphilones can significantly inhibit the release of NO in LPS-induced RAW264.7 macrophages [17], but the underlying molecular mechanism is unclear. In this study, we first examined the anti-inflammatory activity of compounds **1**–**3**. As shown in Table S2, **3** exhibited the most significant activity among them. Therefore, the anti-inflammatory activity of **3** was deeply investigated. To evaluate whether **3** has a cytotoxic activity to RAW264.7 cells, cells were co-incubated with different concentrations of **3** (6.25, 12.5, 25, 50, 100 µM) for 24 h. The results showed that **3** did not exhibit a significant cytotoxic effect under the concentration of 50 µM (Figure 2A). Therefore, the concentration range of 6.25–50 µM was considered valuable in subsequent investigations.

NO has been identified as a pro-inflammatory molecule and is associated with chronic inflammatory diseases and cancers [26,27]. NO production is generally regarded as a marker of macrophage activation, which is closely related to the pathogenesis of inflammatory diseases [28]. In the following study, we first tested whether **3** can affect the NO production of RAW264.7 cells. As shown in Figure 2B, compound **3** significantly suppressed LPS-induced NO production in a dose-dependent way. NO contents were 49.49, 43.70, 35.48, and 20.45 µM when treated with 6.25, 12.5, 25.0, and 50.0 µM of compound **3**, respectively.

### 2.3. Compound ***3*** Reduced LPS-Induced over Expression of COX-2, IL-6, IL-1β, and iNOS

**Figure 3:**
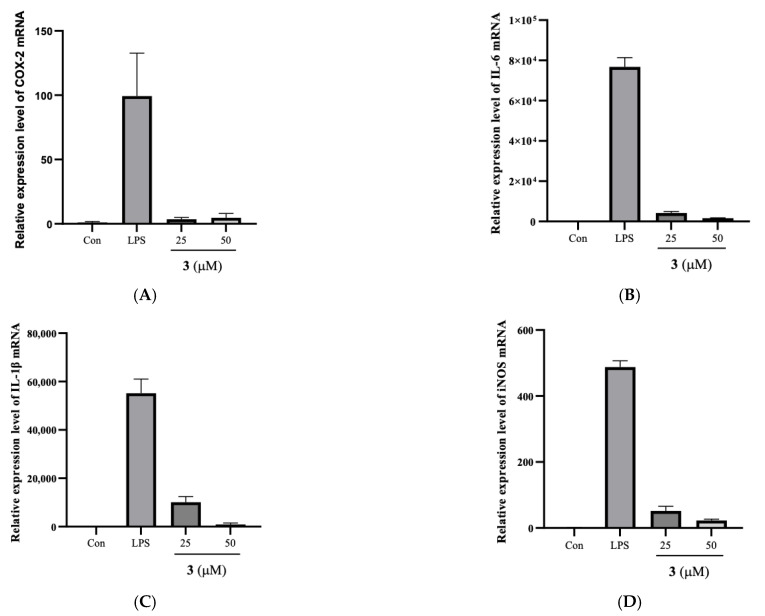
Effects of **3** on the mRNA expressions of COX-2, IL-6, IL-1β, and iNOS in LPS-induced RAW264.7 cells. (**A**–**D**) Cells were plated in cell culture plates at a density of 1 *×* 10^7^ cells/well and incubated with **3** and LPS. After preparation of the nuclear fraction, qRT-PCR was performed to measure the mRNA expression levels of inducible COX-2, IL-6, IL-1β, and iNOS.

As a main inflammatory mediator, NO is synthesized in response to inflammatory stimuli [29,30]. It is reported that a high level of NO not only promotes the inflammatory response but also increases oxidative stress [31,32]. The COX enzymes transform arachidonic acid into prostaglandins, promoting oxidative stress and chronic inflammatory conditions [33]. Thus, down-regulation of these inflammatory intermediaries provides a potential therapeutic strategy for modulating inflammation-related disorders. To further reveal the anti-inflammatory activity of **3**, RAW264.7 cells were pretreated with **3** for 30 min and then stimulated with LPS for 6 h. The transcriptional expression levels of these cytokines and inflammatory enzymes were measured by RT-PCR, and the results demonstrated that the stimulation of RAW 264.7 macrophages with LPS notably elevated the mRNA levels of COX-2 (Figure 3A), IL-6 (Figure 3B), IL-1β (Figure 3C), and iNOS (Figure 3D). Compound **3** significantly attenuated the mRNA levels of pro-inflammatory (iNOS and COX-2) and cytokines (IL-6 and IL-1β).

### 2.4. Effect of 3 on NF-κB Translocation

**Figure 4:**
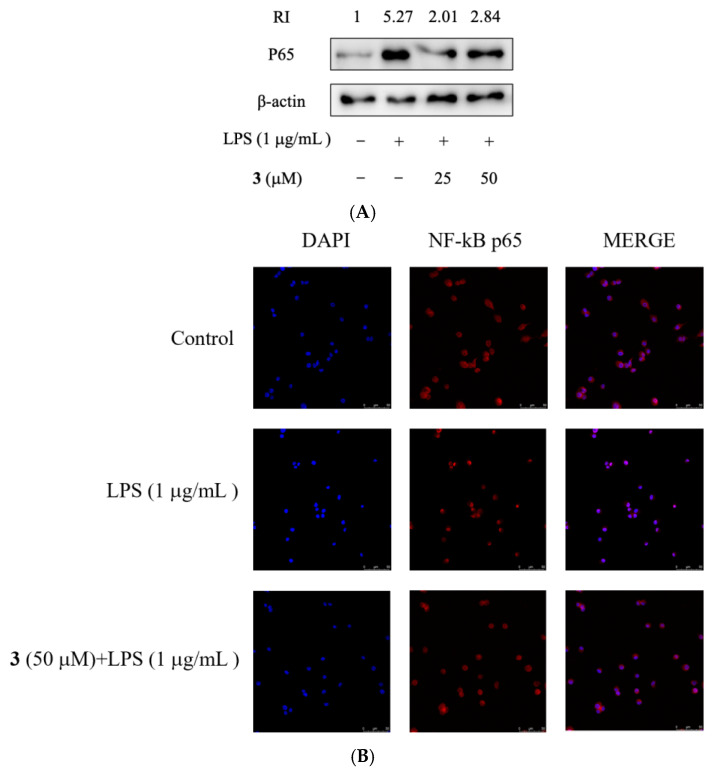
Effects of compound **3** on translocation of transcription factors in LPS-induced RAW264.7 cells. (**A**) Cells (1 × 10^7^ cells) were incubated with various concentrations of **3** (25 µM and 50 µM) and LPS for the indicated time. The protein expression levels of p65 were measured by Western blot analysis. (**B**) Confocal microscopy was visualized for RAW 264.7 cells which were pre-treated with **3** and LPS.

The nuclear translocation of NF-κB transcriptional subunit p65 is a crucial procedure for the activation of most pro-inflammatory cytokines, including IL-1β, IL-6, and TNF-α [34,35]. LPS can induce inflammation by triggering transcriptional activation of inflammation-inducing genes through the migration of NF-κB to the nucleus [36]. To evaluate if NF-κB signaling could be regulated by **3**, the nuclear protein levels of p65 were assayed in RAW264.7 cells treated with different concentrations of **3**. As shown in Figure 4A, LPS up-regulated p65 levels in the RAW264.7 cell nucleus, which were inhibited, especially in the nucleus, by **3**. Furthermore, immunofluorescent staining demonstrated that NF-κB p65 is mainly distributed in the cytoplasm. After LPS treatment, the majority of intracellular p65 translocated to the nucleus, as indicated by the strong nuclear staining for NF-κB p65 (Figure 4B). However, pre-treatment of **3** significantly reduced the p65 nuclear accumulation (Figure 4B). Therefore, treatment of **3** prevented the translocation of NF-κB in RAW264.7 cells induced by LPS.

### 2.5. Compound ***3*** Suppressed NF-κB Activity Induced by LPS 

**Figure 5:**
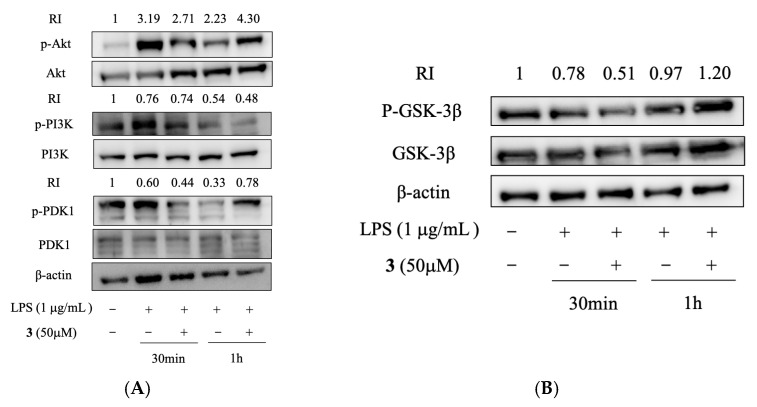
Effects of compound **3** on PI3K/Akt signaling pathway in LPS-induced RAW264.7 cells. (**A**,**B**) Cells (1 × 10^7^ cells) were pre-treated with **3** and then incubated with LPS for the indicated time. After preparation of the total protein, the phosphorylation levels of Akt, PI3K, PDK1, and GSK-3β were determined by Western blot.

The PI3K/Akt pathway has been documented to positively regulate NF-κB and Toll-like receptor-mediated proinflammatory responses in LPS-stimulated RAW 264.7 cells [37,38]. We, therefore, examined the potency of **3** on the PI3K/Akt signaling pathway, and the results showed that the phosphorylation of Akt, PI3K, and PDK1 induced by LPS were significantly suppressed by **3** in RAW264.7 cells (Figure 5A). The rapid phosphorylation of GSK-3β is another important step for the accumulation of p65 in the nucleus, so the phosphorylation level of GSK-3β was also determined. The result showed that **3** significantly suppressed the phosphorylation level of GSK-3β (Figure 5B), thus inhibiting the activation of NF-κB induced by LPS. This result suggested that the protection of **3** against LPS-induced inflammation should rely on its inhibition of NF-κB activation.

Penazaphilones J–L belong to the sclerotiorin type of azaphilone. A previous study showed that sclerotiorin derivatives exhibit various bioactivities. Isochromophilones I and II were novel gp120-CD4 binding inhibitors and potential lead compounds for HIV therapy [39]. Sclerotiorin and isochromophilone IV showed antitumor activity by inhibiting the interaction of Grb2−Shc and bacteriostatic activity against all Gram-positive and Gram-negative bacteria [40,41]. Sclerotiorin is also an inhibitor of lipoxygenase [42]. Isochromophilones III−V and isochromophilone VI were potent inhibitors of acylCoA:cholesterol acyltransferase, promising inhibition sites for the treatment of atherosclerosis and hypercholesterolemia [43]. In this study, we found that penazaphilone L exhibited the most significant anti-inflammatory activity without obvious cytotoxicity. Therefore, we disclosed the underlying mechanism of its anti-inflammation that penazaphilone L suppressed the mRNA expression levels of COX-2, IL-6, IL-1β, and iNOS, inhibited the phosphorylation of PI3K, PDK1, Akt, and GSK-3β, thus inhibiting translocation of NF-κB and resulting in the prevention of LPS-induced inflammation. 

## 3. Materials and Methods

### 3.1. Chemicals and Reagents

See Supplementary Materials.

### 3.2. Strain and Fermentation Conditions

*P. sclerotiorum* cib-411 was preserved in Chengdu Institute of Biology, CAS. The medium and fermentation procedures were the same as those reported [17].

### 3.3. Extraction and Isolation

The fermented rice culture (20 Kg) was soaked with CH_3_OH (60 L) at 60 °C three times (24 h each time). The filtered solvent was condensed under reduced pressure to yield an extract. The extract was loaded on a silica gel column eluted with CH_2_Cl_2_/CH_3_OH (20:1, 10:1, 5:1, 3:1, 1:1, 0:1, *v*/*v*) to give eight fractions (fractions A–H). Fraction D was further fractionated over a silica gel column eluted with petroleum ether/acetone (5:1, 2:1, *v*/*v*) to afford compounds **7** (52 mg), **8** (86 mg), and **9** (150 mg). Separation of fraction E on an RP-18 silica gel column with CH_3_OH/H_2_O as eluent (20:80, 40:60, 60:40, 80:20, 100:0, *v*/*v*) gave eight subfractions (E1-E8). Subfraction E3 was further fractionated by semipreparative HPLC with CH_3_CN/H_2_O (0.8 mL/min, 33:67, *v*/*v*) to afford **2** (4.9 mg, t*_R_* = 20.9 min) and **3** (4.0 mg, t*_R_* = 16.9 min). Fraction F was separated over a Sephadex LH-20 column to yield five subfractions (F1–F5). Compound **1** (6.9 mg, t*_R_* = 11.1 min) was obtained from subfraction F4 by HPLC (CH_3_CN/H_2_O, 0.8 mL/min, 35/65, *v*/*v*). Subfraction E6 was subjected to preparative HPLC (CH_3_CN/H_2_O/CH_3_COOH, 10 mL/min, 60/39/1, *v*/*v*/*v*) to yield compounds **5** (180 mg, t*_R_* = 10.1 min), **4** (230 mg, t*_R_* = 18.5 min), and **6** (150 mg, t*_R_* = 21.5 min).

### 3.4. Structure Identification

#### 3.4.1. Spectrometric Analyses

See Reference [17].

#### 3.4.2. Penazaphilone J (**1**)

*Penazaphilone J* (**1**): Red amorphous powder; [α${]}_{20}^{D}$ −141° (*c* 0.0135, MeOH); UV (MeOH): λ_max_ (log *ε*) 474 (3.36), 371 (4.12), 224 (4.03); HRESIMS *m*/*z* 588.1759 [M + H]^+^, (calcd for C_26_H_36_ClNO_10_P, 588.1759). ^1^H and ^13^C NMR data are in Table 1.

#### 3.4.3. Penazaphilone K (**2**)

*Penazaphilone K* (**2**): Red amorphous powder; [α${]}_{20}^{D}$ −157° (*c* 0.0185, MeOH); UV (MeOH): λ_max_ (log *ε*) 480 (3.46), 372 (4.72), 221 (4.24); HRESIMS *m*/*z* 519.2256 [M + H]^+^, (calcd for C_27_H_36_ClN_2_O_6_, 519.2254). ^1^H and ^13^C NMR data are in Table 1.

#### 3.4.4. Penazaphilone L (**3**)

*Penazaphilone L* (**3**): Red amorphous powder; [α${]}_{20}^{D}$ −91° (*c* 0.0165, MeOH); UV (MeOH): λ_max_ (log *ε*) 479 (3.28), 372 (4.09), 227.5 (4.04); HRESIMS *m*/*z* 505.2097 [M + H]^+^, (calcd for C_26_H_34_ClN_2_O_6_, 505.2099). ^1^H and ^13^C NMR data are in Table 1.

### 3.5. Anti-Inflammatory Activity Assays

#### 3.5.1. Cell Line and Cell Culture

The RAW264.7 macrophage cells were provided by the American Type Culture Collection (Rockville, MD, USA). Cells were cultured in RPMI 1640 medium supplemented with 10% FBS and 1% antibiotics (100 U/mL penicillin and 100 µg/mL streptomycin), and incubated at 37 °C in a humidified incubator with 5% CO_2_ (SANYO, Tokyo, Japan).

#### 3.5.2. Cell Viability Assay

The cytotoxicity against RAW264.7 cells was evaluated by MTT method [44]. Concisely, 96-well plates (Corning, Suzhou, China) containing cells at a density of 1 × 10^5^ cells/well were incubated at 37 °C overnight, then treated with different compounds (0, 6.25, 12.5, 25, 50, 100 µM) for 24 h. The medium was decanted, and the MTT (0.5 mg/mL) working solution was dripped into each well and stained for an extra 4 h at 37 °C in the dark. Subsequently, the formazan crystals were dissolved by adding a stopping buffer. After thorough dissolution, the absorbance at 550 nm was measured by an Infinite M200 PRO microplate reader (Tecan, Männedorf, Switzerland) to determine the product amount.

#### 3.5.3. Measurement of Nitric Oxide (NO)

NO content of the supernatant was checked by Griess reagent (1% sulfanilamide in 5% phosphoric acid and 0.1% N-1-naphthylenediamine dihydrochloride), similar to our previous publication [44]. In brief, RAW264.7 cells in a 96-well plate (1 *×* 10^5^ cells/well) were incubated overnight. Cells were treated with different concentrations of samples (30 min) first, then incubated with LPS (1 µg/mL) for 6 h. After incubation, the cell supernatant (100 µL) was moved to another 96-well plate and commingled with Griess reagent (*v*/*v*, 1:1) for 10 min incubation at indoor temperature. The absorbance was determined at 570 nm. 

#### 3.5.4. Quantitative RT-PCR

Cells were pre-treated with different concentrations of compound **3** for 30 min and then incubated with LPS (1 µg/mL) for 6 h. After being washed by PBS, total RNA was extracted with Trizol. Next, cDNA was synthesized by the RevertAid First Strand cDNA Synthesis Kit. The primers were used for the amplification of GAPDH, COX-2, iNOS, IL-6, and IL-1*β*. The semi-quantitative PCR amplified products of GAPDH were separated by 1% agarose gel electrophoresis, stained with GoldView (Solarbio, Beijing, China), and photographed using a Gel Doc XR+ with Image Lab Software (Bio-Rad Laboratories, Hercules, CA, USA). The expression levels of COX-2 mRNA, IL-6 mRNA, IL-1*β* mRNA, and iNOS mRNA were checked by qRT-PCR analysis with SYBR Green Premix Ex Taq (Roche, Mannheim, Germany) and performed by the CFX96 Touch Real-Time PCR Detection System (Bio-Rad, Hercules, CA, USA). The mRNA expression levels were analyzed using the 2^−ΔΔCT^ method. The results were normalized to GAPDH expression and quantified relative to the corresponding mRNA expression in the control group, which was normalized to 1 [44].

#### 3.5.5. Western Blot

Cells were pre-treated with **3** at different concentrations for 30 min and then incubated with LPS (1.0 µg/mL) for different time points. The cells were washed with cold PBS, and then scraped out. The total protein and nuclear protein were respectively obtained using RIPA lysis buffer and Nuclear and Cytoplasmic Extraction Kit (CWBio, Beijing, China). The protein concentrations were determined by using the BCA protein assay kit (Absin, Shanghai, China). The protein samples were put into a gel loading buffer and boiled at 95 °C for 5 min. After separating on a 10% SDS-polyacrylamide gel for 2 h, the protein was transferred to PVDF membranes and blocked with 5% BSA for 2 h at room temperature, incubated overnight with primary antibodies at 4 °C, and washed three times with the 0.1% TTBS for 10 min. After that process, the secondary antibodies with horseradish peroxidase (HRP)-conjugated IgG were further incubated for 2 h at room temperature (1:2000 dilution). With an enhanced chemiluminescence detection system (Tanon 5200 Multi, Beijing, China), the blots were visualized using an eECL kit. Subsequently, the protein bands were quantified using image J [44].

#### 3.5.6. In Vitro Cell Uptake

RAW264.7 cells in 24-well plates (1 × 10^6^ cells/well) were incubated at 37 °C with 5% CO_2_ overnight, then pre-treated with different concentrations of **3** for 30 min, and later incubated with LPS (1 µg/mL) for 6 h. After washing with cold PBS, the cells were fixed with 4% paraformaldehyde for 15 min, blocked with 3% BSA for 1 h, and finally incubated with the primary antibody NF-κB p65 (1:400) for 1 h. After being rinsed with PBS three times, cells were incubated with secondary antibodies conjugated with Alexa Fluor 488 for an extra 2 h. Coverslips were then mounted with DAPI containing mounting medium. Immunofluorescent images were captured using a confocal laser scanning microscope (Leica TCS SP8, Leica Microsystem, Bensheim, Germany) [44].

#### 3.5.7. Data Analysis

SPSS software (SPSS Inc., Chicago, IL, USA) was employed for statistical analysis. The results are expressed as the mean *±* SD. The statistical significance of the difference was analyzed by one-way ANOVA followed by Duncan’s multiple range test, and *p* < 5% were considered statistically.

## 4. Conclusions

In a summary, this study disclosed three new hydrophilic azaphilone alkaloids (**1**–**3**) from the fungus *P. sclerotiorum*. Compound **1** is a structurally intriguing natural pigment and represents a novel azaphilone alkaloid containing a glycerophosphate choline moiety. Compounds **2** and **3**, respectively, contain lysine and an ornithine moiety, which seldom exist in azaphilone metabolites. Compounds **1**–**3** possessed anti-inflammatory activity. Compound **3** exhibited the most significant bioactivity, suggesting the side chain on N-2 greatly influenced the anti-inflammatory activity. The investigation of the underlying mode of action confirmed that compound **3** might exert anti-inflammatory activity by suppressing the production of nitric oxide (NO); down-regulating the mRNA levels of cyclooxygenase-2 (COX-2), interleukin-6 (IL-6), interleukin-1β (IL-1β), and NO synthase (iNOS); and inhibiting the phosphorylation of PI3K, PDK1, Akt, and GSK-3β, as well as the nuclear translocation of nuclear factor κB (NF-κB) p65, a series of genes and proteins in the NF-κB signaling pathway. The results in this study suggested that penazaphilone L (**3**) is a potential functional colorant and may have a promising prospect in food industry.

## Figures and Tables

**Table 1:** NMR Spectroscopic Data of **1**–**3** in CD_3_OD (*δ* in ppm, *J* in Hz).

No.	1		2		3	
	*δ*_H_	*δ*_C_	*δ*_H_	*δ*_C_	*δ*_H_	*δ*_C_
1	8.24 (1H, s)	144.6	8.19 (1H, s)	143.7	8.21 (1H, s)	143.7
3		148.3		148.2		148.1
4	7.18 (1H, s)	112.6	7.18 (1H, s)	112.6	7.19 (1H, s)	112.6
4a		151.9		151.3		151.2
5		101.3		101.3		101.4
6		185.6		185.5		185.6
7		86.2		86.2		86.2
8		194.9		195.0		195.0
8a		116.4		116.6		116.6
9	6.58 (1H, d, 15.5)	117.1	6.47 (1H, d, 15.5)	116.6	6.48 (1H, d, 15.3)	116.4
10	7.11 (1H, d, 15.4)	146.8	7.13 (1H, d, 15.4)	146.6	7.15 (1H, d, 15.4)	146.8
11		134.0		133.8		133.9
12	5.78 (1H, d, 9.7)	148.8	5.80 (1H, d, 9.7)	149.1	5.81 (1H, d, 9.8)	149.1
13	2.54 (1H, m)	36.2	2.55 (1H, m)	36.2	2.55 (1H, m)	36.2
14	1.45 (1H, m) 1.37 (1H, m)	31.1	1.46 (1H, m) 1.36 (1H, m)	31.1	1.46 (1H, m) 1.38 (1H, m)	31.1
15	0.90 (3H, t, 7.4)	12.4	0.90 (3H, t, 7.4)	12.3	0.90 (3H, t, 7.3)	12.4
16	1.03 (3H, d, 6.6)	20.6	1.04 (3H, d, 6.6)	20.6	1.04 (3H, d, 6.6)	20.5
17	1.94 (3H, s)	12.8	1.93 (3H, s)	12.8	1.95 (3H, s)	12.8
18	1.52 (3H, s)	23.8	1.50 (3H, s)	23.8	1.50 (3H, s)	23.8
19		171.6		171.6		171.6
20	2.12 (3H, s)	20.2	2.12 (3H, s)	20.2	2.12 (3H, s)	20.2
1′	4.42 (2H, m)	55.6	8.11 (1H, s)	164.8	8.09 (1H, s)	164.7
2′	4.16 (2H, m)	64.5	3.67 (1H, m)	55.2	3.68 (1H, m)	55.0
3′	3.79 (2H, m)	67.8	1.83 (2H, m)	31.6	1.35 (1H, m) 1.29 (1H, m)	28.8
4′	3.71 (1H, m)	72.5	1.46 (1H, m) 1.36 (1H, m)	23.0	1.88 (2H, m)	27.1
5′	3.52 (2H, m)	63.7	1.83 (2H, m)	30.6	4.21 (2H, m)	54.9
6′			4.16 (2H, m)	55.4		

The ^1^H NMR spectra of **1**, **2**, and **3** were measured at 400 MHz. The ^13^C NMR spectrum of **1** was acquired at 100 MHz, and **2** and **3** were acquired at 150 MHz.

## Data Availability

Not applicable.

## Supplementary Materials

The following supporting information can be downloaded at: https://www.mdpi.com/article/10.3390/molecules28073146/s1, Figure S1: The HRESIMS spectrum of penazaphilone J (**1**); Figure S2: The 1H NMR spectrum of penazaphilone J (**1**); Figure S3: The 13C NMR spectrum of penazaphilone J (**1**); Figure S4: The DEPT135 spectrum of penazaphilone J (**1**); Figure S5: The 31P NMR spectrum of penazaphilone J (**1**); Figure S6: The HSQC spectrum of penazaphilone J (**1**); Figure S7: The HMBC spectrum of penazaphilone J (**1**); Figure S8: CD spectrum of penazaphilone J (**1**); Figure S9: The HRESIMS spectrum of penazaphilone K (**2**); Figure S10: The 1H NMR spectrum of penazaphilone K (**2**); Figure S11: The 13C NMR spectrum of penazaphilone K (**2**); Figure S12: The DEPT135 spectrum of penazaphilone K (**2**); Figure S13: The HSQC spectrum of penazaphilone K (**2**); Figure S14: The HMBC spectrum of penazaphilone K (**2**); Figure S15: CD spectrum of penazaphilone K (**2**); Figure S16: The HRESIMS spectrum of penazaphilone L (**3**); Figure S17: The 1H NMR spectrum of penazaphilone L (**3**); Figure S18: The 13C NMR spectrum of penazaphilone L (**3**); Figure S19: The DEPT135 spectrum of penazaphilone L (**3**); Figure S20: The HSQC spectrum of penazaphilone L (**3**); Figure S21: The HMBC spectrum of penazaphilone L (**3**); Figure S22: CD spectrum of penazaphilone L (**3**); Table S1: The sequences of primers for qRT-PCR; Table S2: The preliminary screening results of nitric oxide inhibitory effects of compounds **1**–**3**.

## Sample Availability

Samples of the compounds **1**–**3** are available from the authors.
