# Supplementary material for: Penazaphilones J–L, Three New Hydrophilic Azaphilone Pigments from Penicillium sclerotiorum cib-411 and Their Anti-Inflammatory Activity
Source: Molecules. 2023 Mar 31;28(7):3146. doi: 10.3390/molecules28073146 (PMC10095951; doi:10.3390/molecules28073146)
Supplement: Supplementary file 1 [file molecules-28-03146-s001.zip › molecules-2237760-supplementary.pdf]

Supporting information

Penazaphilones J–L, Three New Hydrophilic Azaphilone Pigments from *Penicillium sclerotiorum cib-411* and Their Anti-Inflammatory Activity

Xia Zhang [1,†], Yeye Hu [2,†], Tao Yang [1,†], Xueqing Qian [1], Weicheng Hu [2,*] and Guoyou Li [1,*]

[1] Key Laboratory of Environmental and Applied Microbiology, Environmental Microbiology Key Laboratory of Sichuan Province, Chengdu Institute of Biology, Chinese Academy of Sciences, Chengdu 610041, China
[2] Institute of Translational Medicine, School of Medicine, Yangzhou University, Yangzhou 225009, China
* Correspondence: hu_weicheng@163.com (W.H.); ligy@cib.ac.cn (G.L.)
† These authors contributed equally to this work.

CONTENTS

Chemical and reagents……………………………………………………………3

Figure S1. The HRESIMS spectrum of penazaphilone J (**1**) 4

Figure S2. The ^{1}H NMR spectrum of penazaphilone J (**1**) 5

Figure S3. The ^{13}C NMR spectrum of penazaphilone J (**1**) 6

Figure S4. The DEPT135 spectrum of penazaphilone J (**1**) 7

Figure S5. The ^{31}P NMR spectrum of penazaphilone J (**1**)...8

Figure S6. The HSQC spectrum of penazaphilone J (**1**)..9

Figure S7. The HMBC spectrum of penazaphilone J (**1**)...10

Figure S8. CD spectrum of penazaphilone J (**1**) 11

Figure S9. The HRESIMS spectrum of penazaphilone K (**2**).....................................12

Figure S10. The ^{1}H NMR spectrum of penazaphilone K (**2**)......................................13

Figure S11. The ^{13}C NMR spectrum of penazaphilone K (**2**).....................................14

Figure S12. The DEPT135 spectrum of penazaphilone K (**2**) 15

Figure S13. The HSQC spectrum of penazaphilone K (**2**) 16

Figure S14. The HMBC spectrum of penazaphilone K (**2**) 17

Figure S15. CD spectrum of penazaphilone K (**2**)...18

Figure S16. The HRESIMS spectrum of penazaphilone L (**3**) 19

Figure S17. The ^{1}H NMR spectrum of penazaphilone L (**3**) 20

Figure S18. The ^{13}C NMR spectrum of penazaphilone L (**3**) 21

Figure S19. The DEPT135 spectrum of penazaphilone L (**3**)......................................22

Figure S20. The HSQC spectrum of penazaphilone L (**3**) 23

Figure S21. The HMBC spectrum of penazaphilone L (**3**)..24

Figure S22. CD spectrum of penazaphilone L (**3**) 25

Table S1 The sequences of primers for qRT-PCR……………………………………26

2.1 Chemicals and reagents

Sephadex LH-20 was from GE Healthcare Bio-Sciences (Uppsala, Sweden). 1-(4,5-dimethylthiazol-2-yl)-3,5-diphenylformazan (MTT), methanol-d_4, sulphanilamide, n-(1-naphthyl) ethylenediamine, dimethyl sulfoxide (DMSO), and lipopolysaccharide (LPS) were purchased from Sigma–Aldrich, St. Louis, MO, USA). The fetal bovine serum (FBS) was from corning (New Zealand), Roswell Park Memorial Institute (RPMI)-1640 was from Invitrogen-Gibco (Beijing, China), and Penicillin/Streptomycin was from Invitrogen-Gibco (Carlsbad, CA, USA). Ambion (Austin, TX, USA) provided Trizol Reagent. CWBio (Jiangsu, China) provided RIPA Lysis Buffer and eECL Western Blot Kit. cDNA synthesis kit and SYBR Green Master were respectively from Thermo Scientific (Waltham, MA, USA) and Roche (Mannheim, Germany). Forward and reverse primers for the targeted genes are synthesized from Sangon Biotech (Shanghai, China). The antibodies used for NF-κB p65 (D14E12; #8242), p-AKt（D9E；#4060), Akt（#9272), p-GSK-3b（D85E12；#5558), GSK-3（D5C5Z；#12456), p-PI3K（#4228), PI3K（19H8；#4257), p-PDK1（C49H2；#3438), and PDK1(#3062) were provided by Cell Signaling Technology (Danvers, MA, USA). Goat anti-rabbit IgG (HRP) and Goat anti-Ms IgG (HRP) were from Abcam (Cambridge, MA, USA). β-actin was provided by CWBio (Beijing, China).

Figure S1. The HRESIMS spectrum of penazaphilone J (**1**)

.	Formula	Neutral mass (Da)	Observed m/z	Mass error (mDa)	Mass error (ppm)	Response	Adducts	Identification status
1	C26H35ClNO10P	587.16871	588.17591	-0.1	-0.1	861178	+H	Identified

Item name: 20200910-ZX-LGY-TJL-17A
Item description:
Channel name: Time 0.0685 +/- 0.0500 minutes

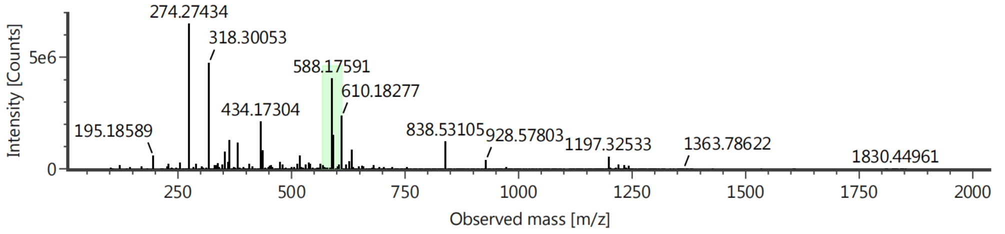

Item name: 20200910-ZX-LGY-TJL-17A
Item description:
Channel name: Time 0.0685 +/- 0.0500 minutes

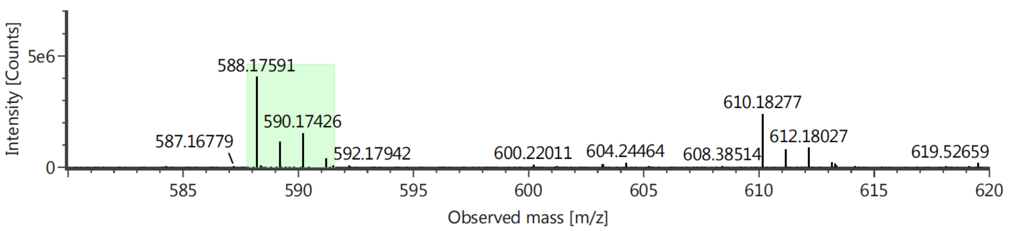

Figure S2. The ^1H NMR spectrum of penazaphilone J (**1**)

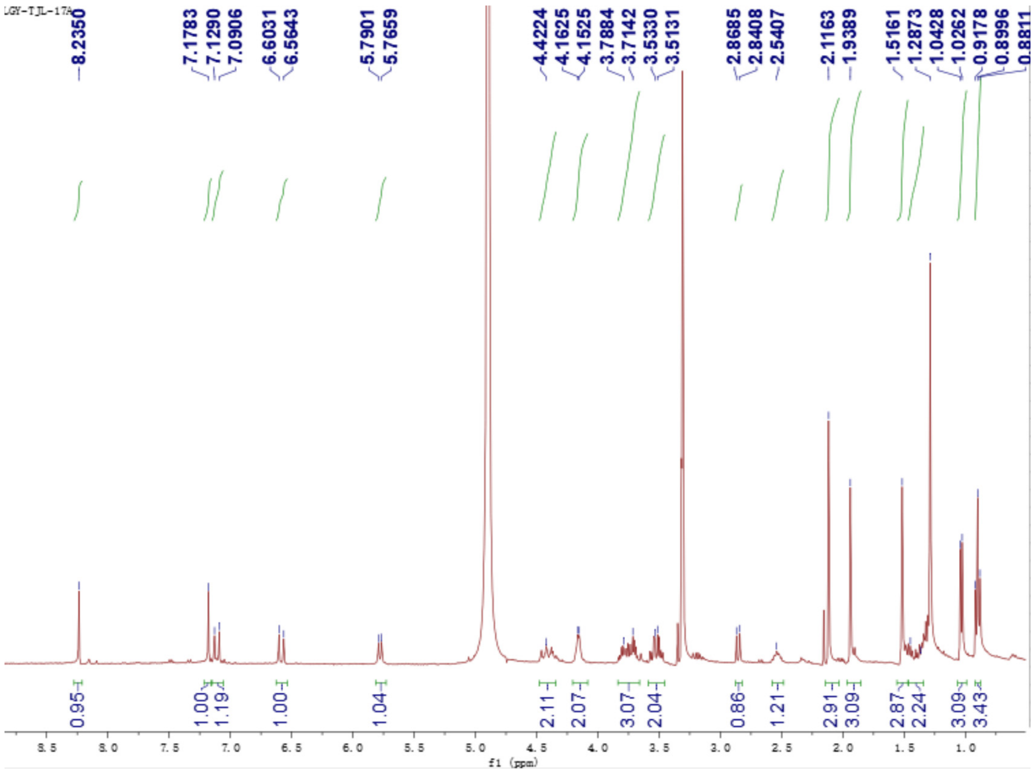

Figure S3. The ^{13}C NMR spectrum of penazaphilone J (**1**)

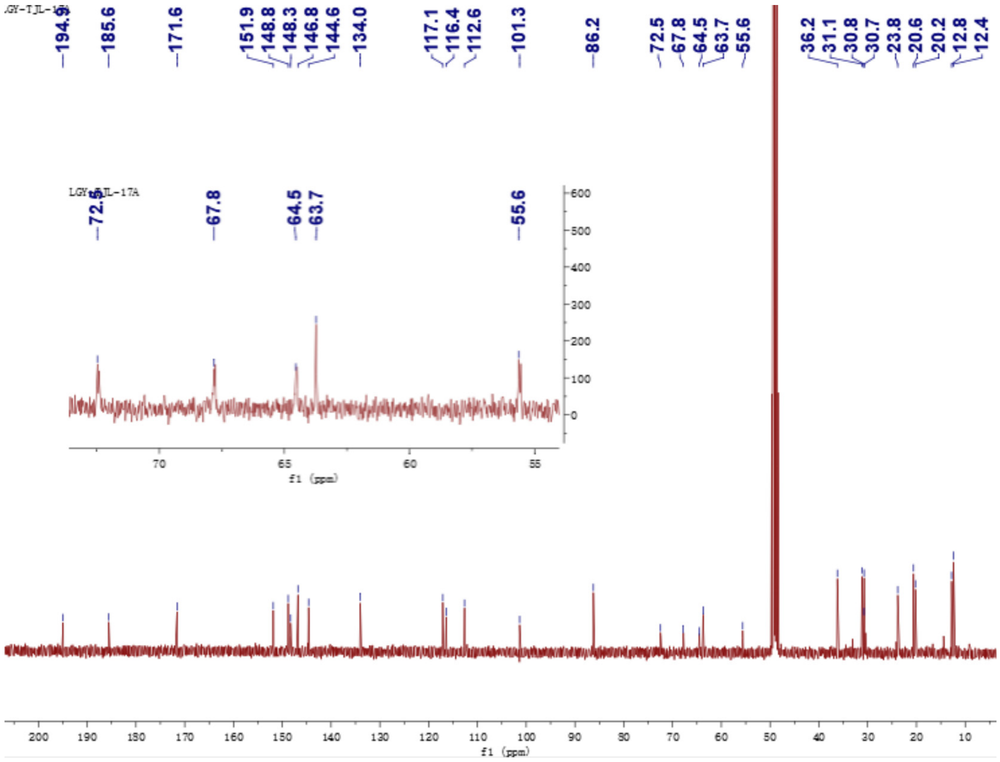

Figure S4. The DEPT135 spectrum of penazaphilone J (**1**)

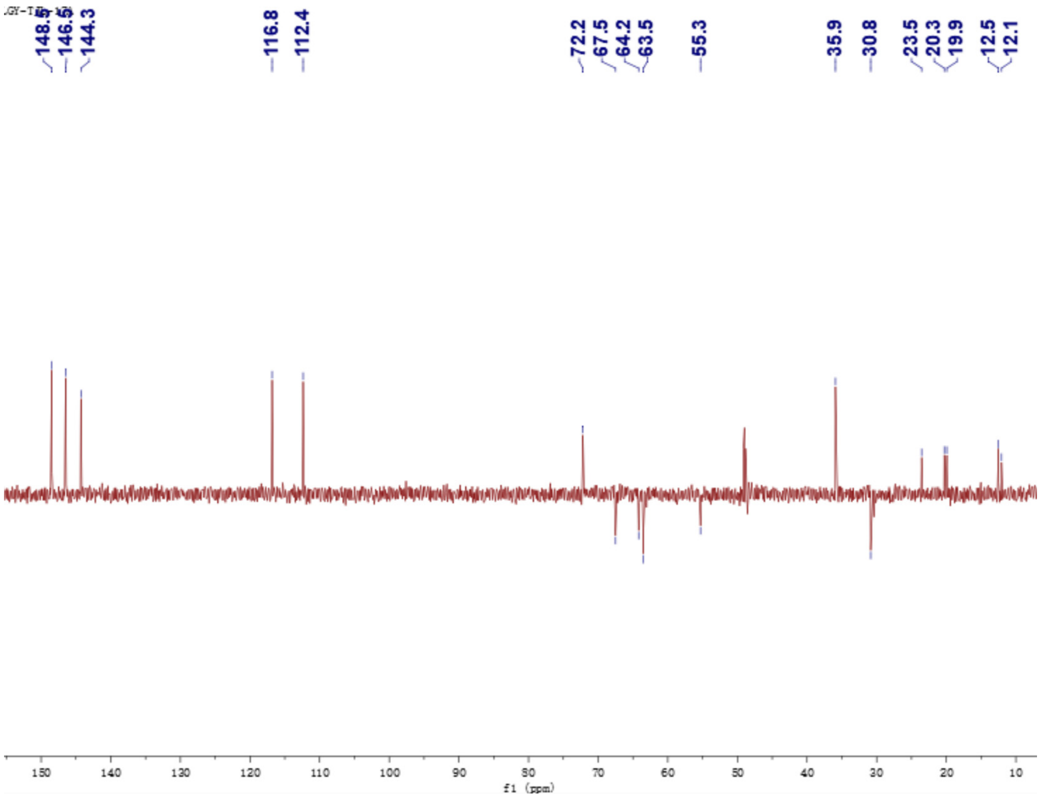

Figure S5. The ^{31}P NMR spectrum of penazaphilone J (**1**)

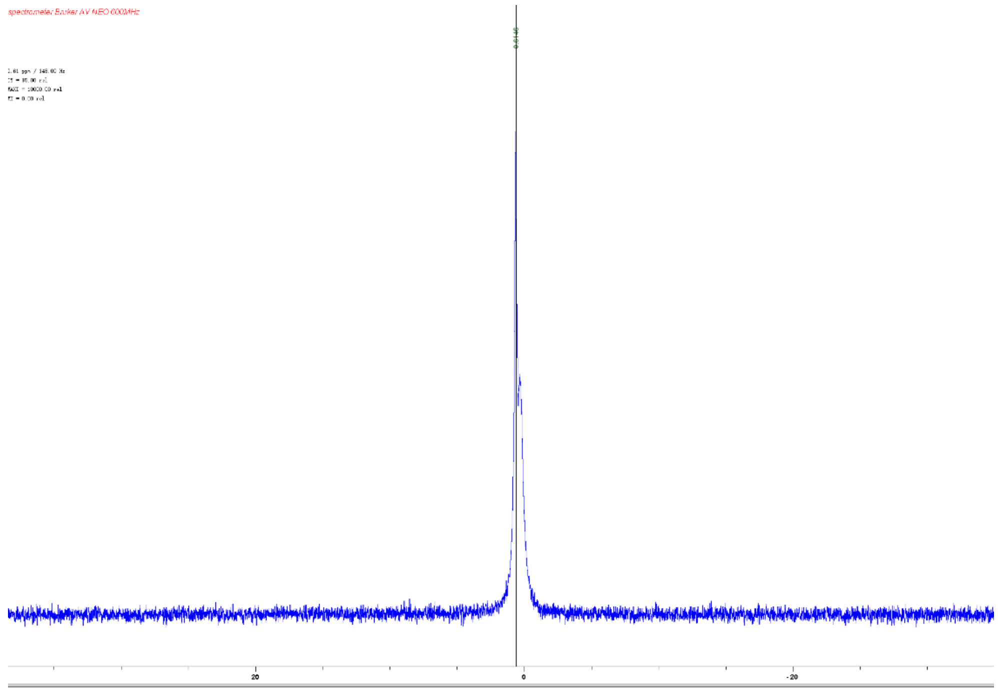

Figure S6. The HSQC spectrum of penazaphilone J (**1**)

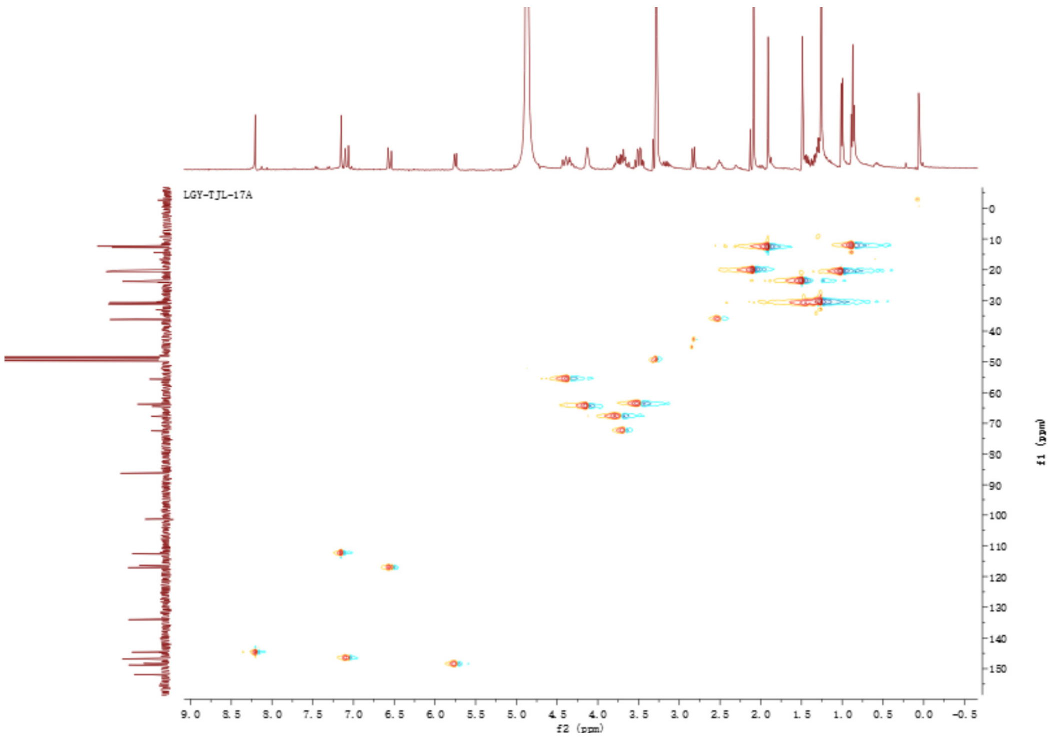

Figure S7. The HMBC spectrum of penazaphilone J (**1**)

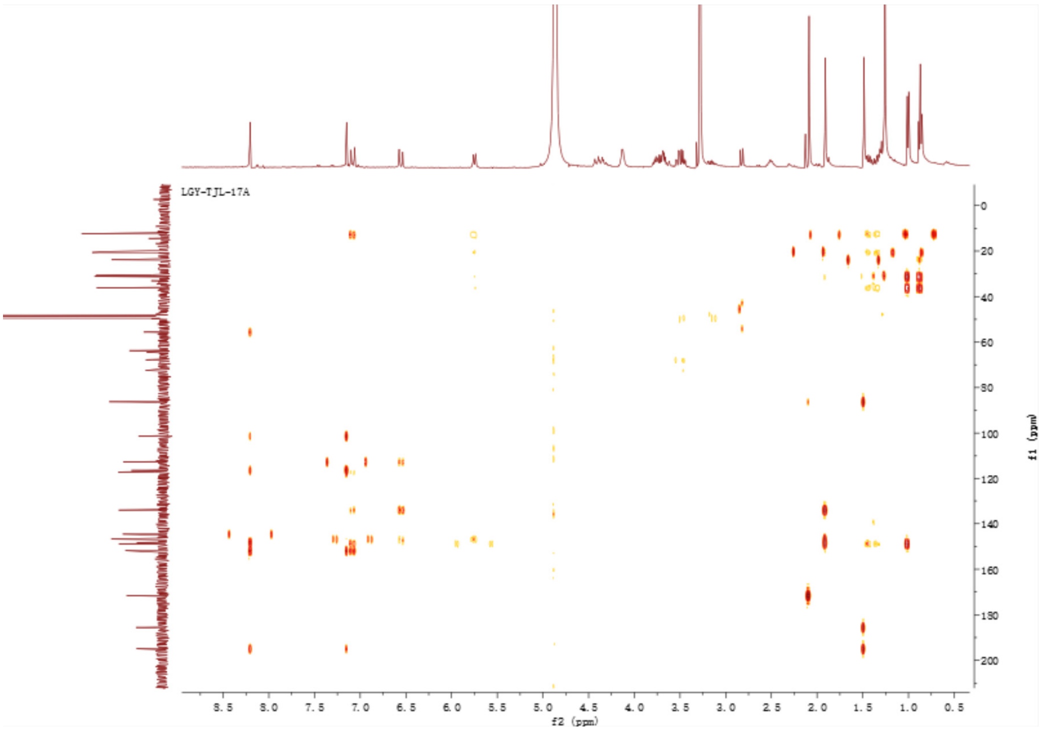

Figure S8. CD spectrum of penazaphilone J (**1**)

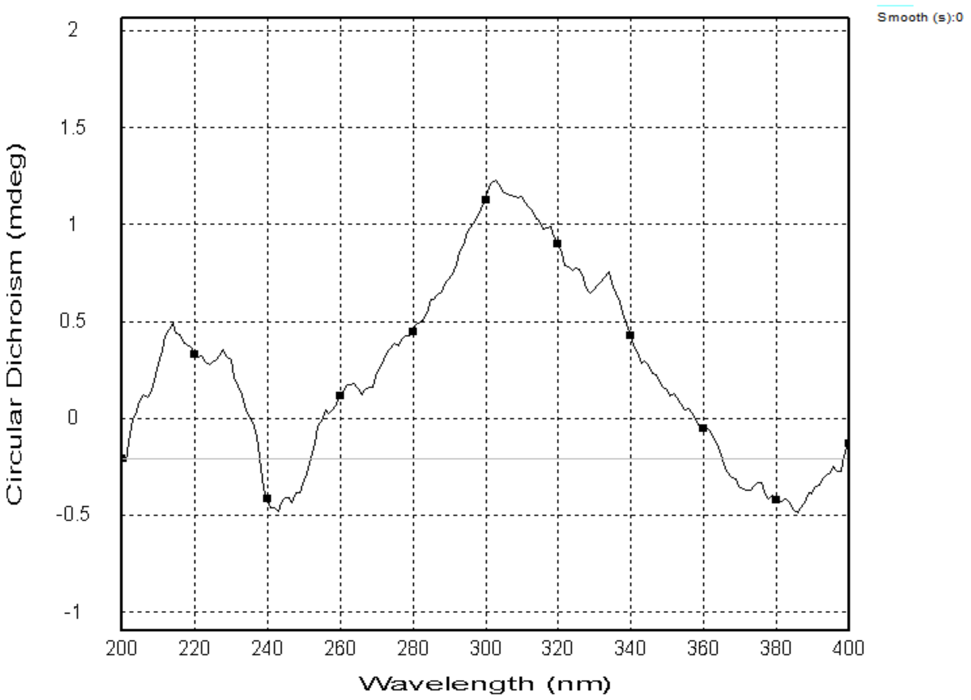

Figure S9. The HRESIMS spectrum of penazaphilone K (**2**)

Item name: 20190404-LGY-TJL-320-2, Sample position: 1:A,7, Replicate number: 1

.	Formula	Neutral mass (Da)	Observed m/z	Mass error (mDa)	Mass error (ppm)	Response	Adducts	Identification status
1	C27H35ClN2O6	518.21836	519.22555	-0.1	-0.2	14291926	+H, +Na	Identified

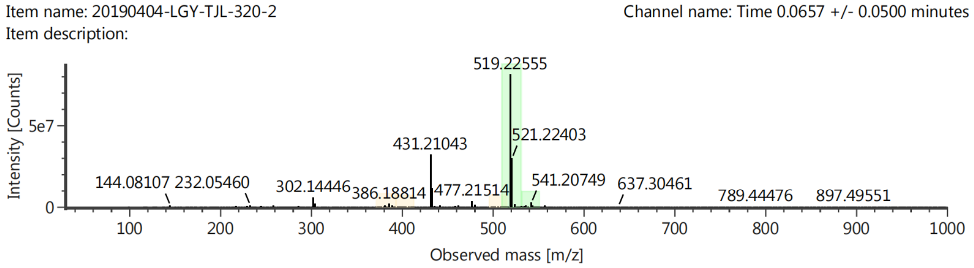

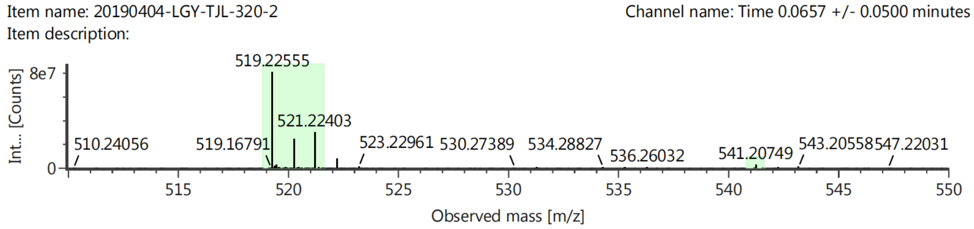

Figure S10. The ^{1}H NMR spectrum of penazaphilone K (**2**)

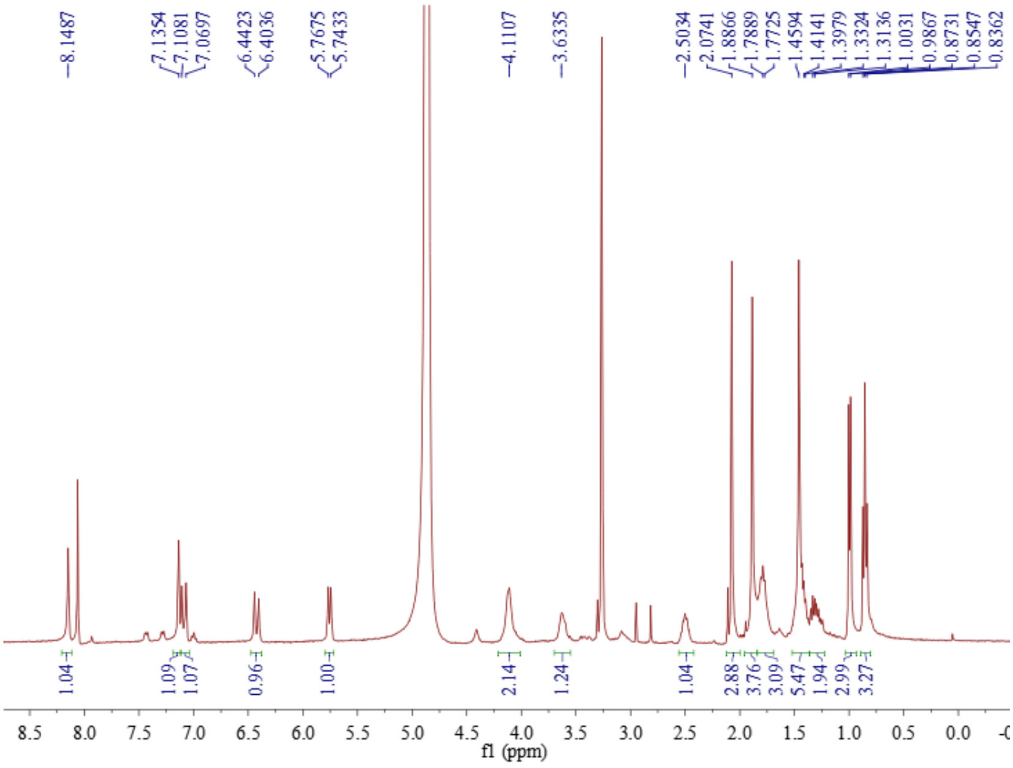

Figure S11. The ^{13}C NMR spectrum of penazaphilone K (**2**)

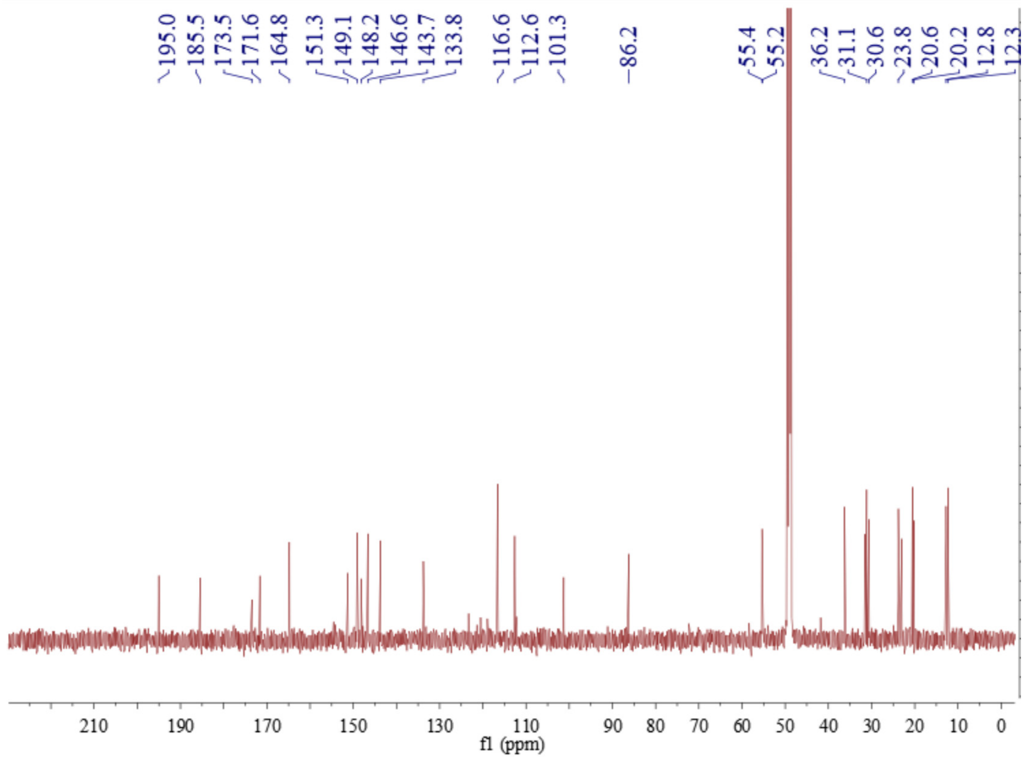

Figure S12. The DEPT135 spectrum of penazaphilone K (**2**)

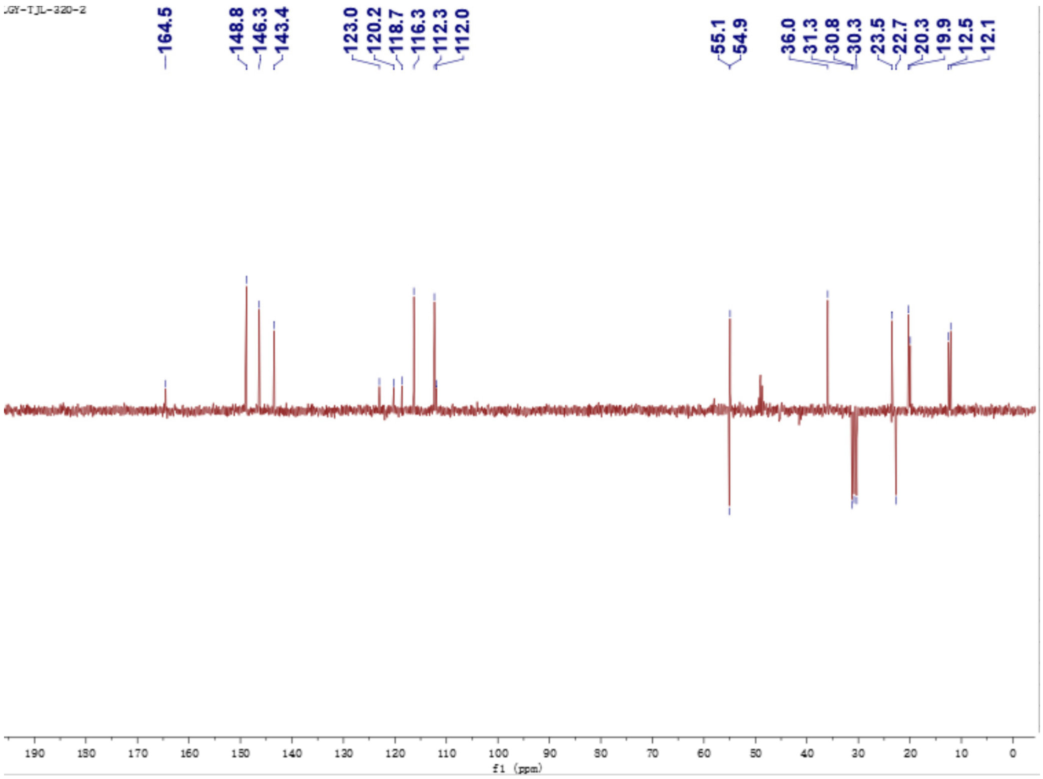

Figure S13. The HSQC spectrum of penazaphilone K (**2**)

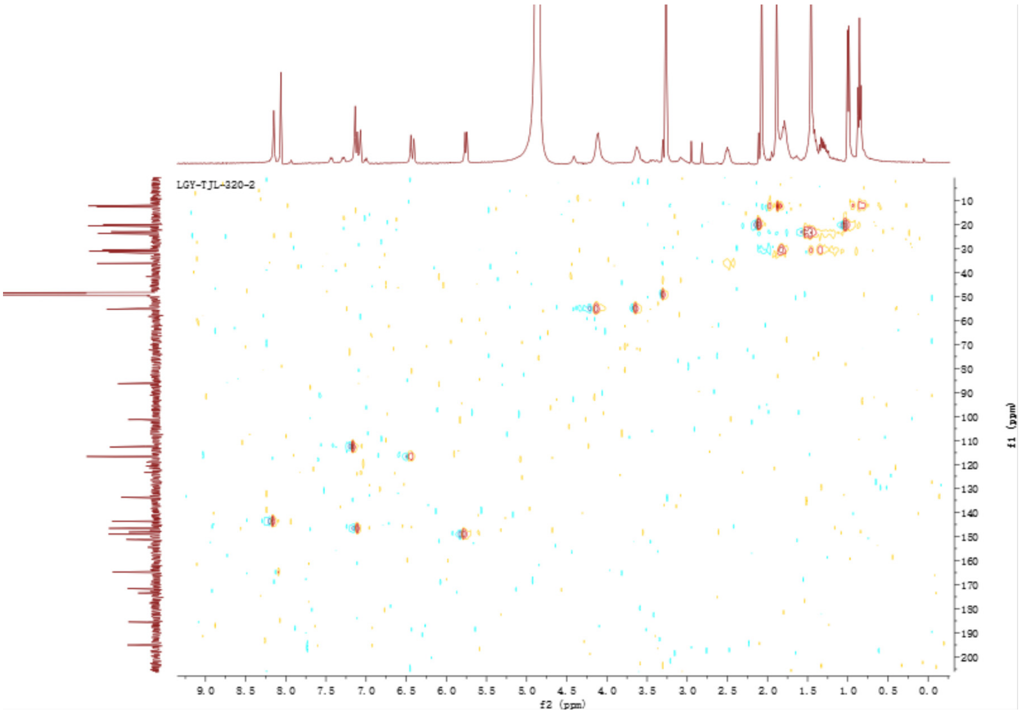

Figure S14. The HMBC spectrum of penazaphilone K (**2**)

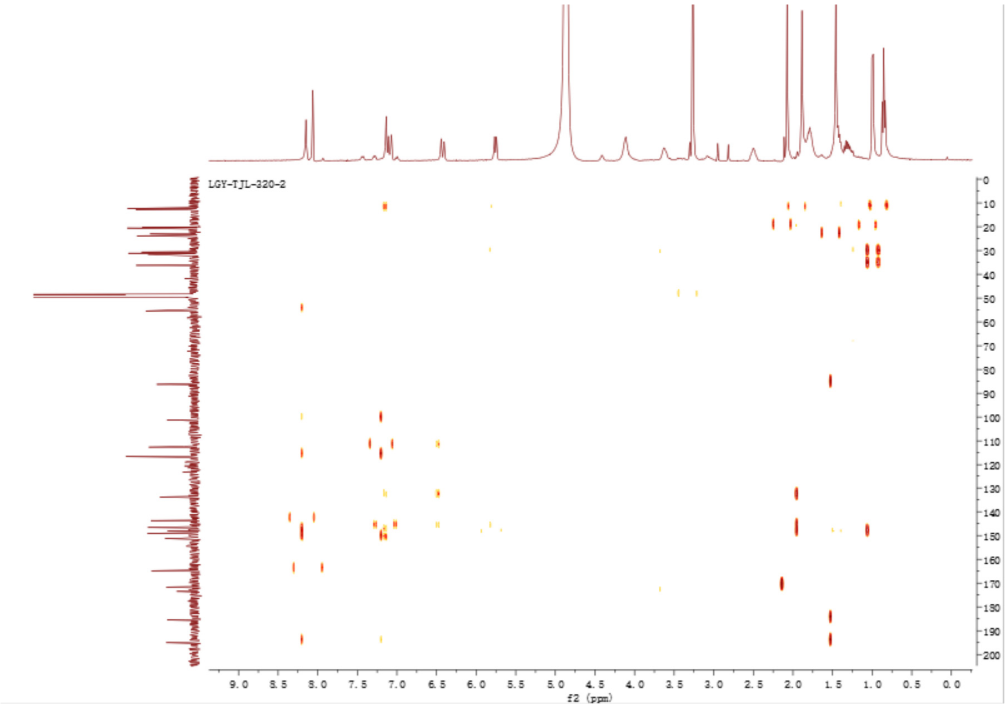

Figure S15. CD spectrum of penazaphilone K (**2**)

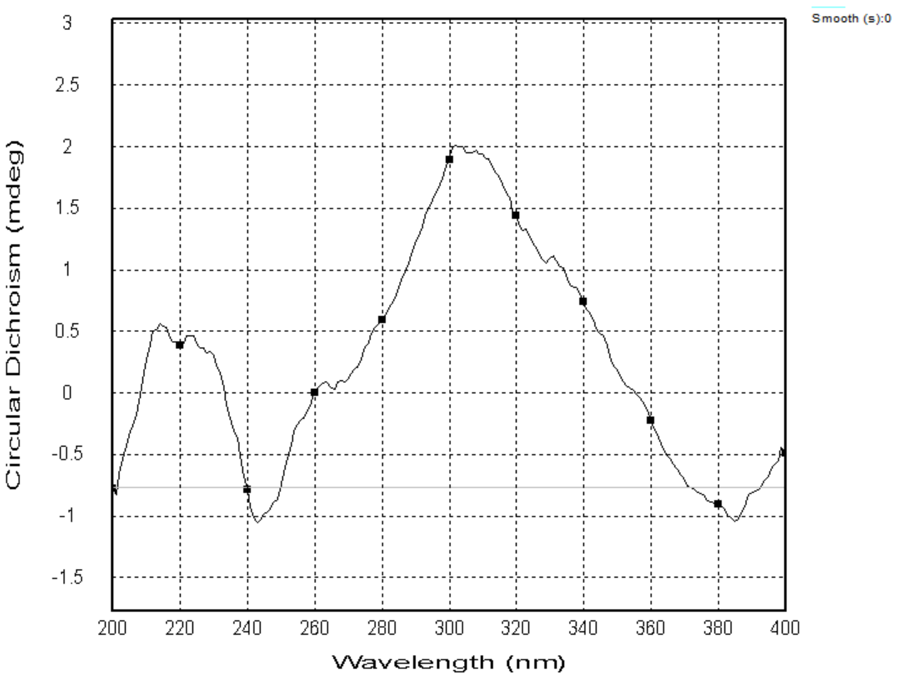

Figure S16. The HRESIMS spectrum of penazaphilone L (**3**)

Item name: 20190404-LGY-TJL-320-1, Sample position: 1:A,6, Replicate number: 1

.	Formula	Neutral mass (Da)	Observed m/z	Mass error (mDa)	Mass error (ppm)	Response	Adducts	Identification status
1	C26H33ClN2O6	504.20271	505.20969	-0.3	-0.6	6439269	+H, +Na	Identified

Item name: 20190404-LGY-TJL-320-1 Channel name: Time 0.0655 +/- 0.1500 minutes

Item description:

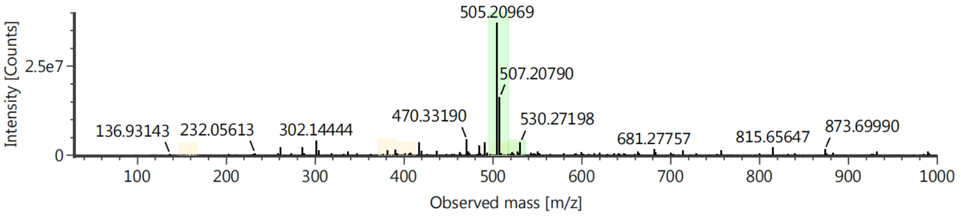

Item name: 20190404-LGY-TJL-320-1 Channel name: Time 0.0655 +/- 0.1500 minutes

Item description:

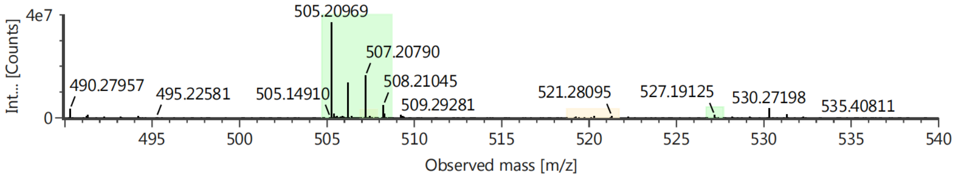

Figure S17. The ^{1}H NMR spectrum of penazaphilone L (**3**)

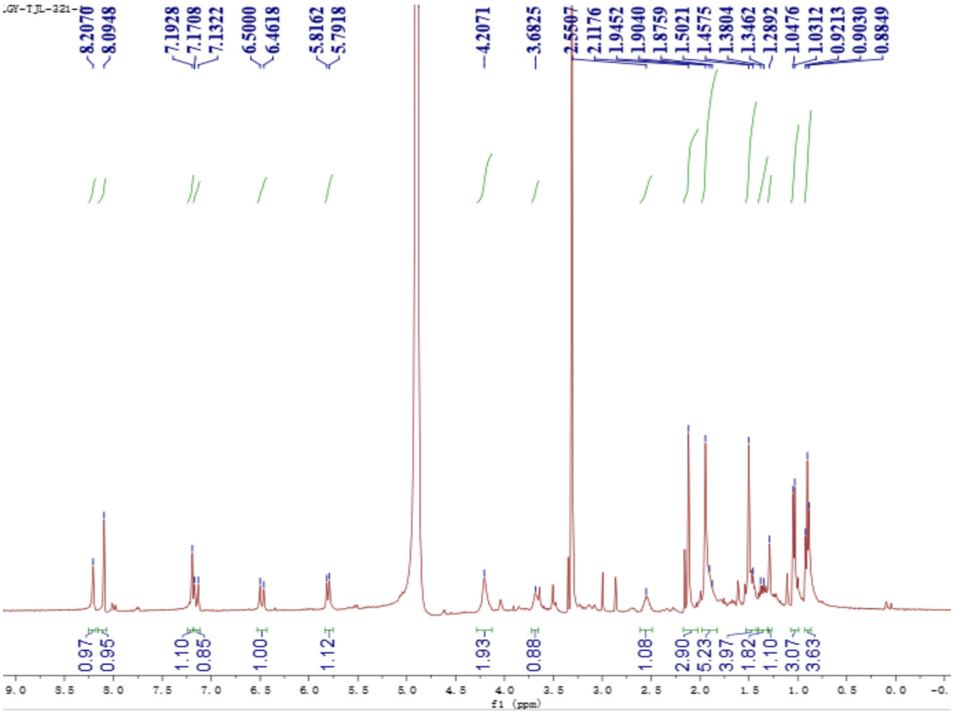

Figure S18. The ^{13}C NMR spectrum of penazaphilone L (**3**)

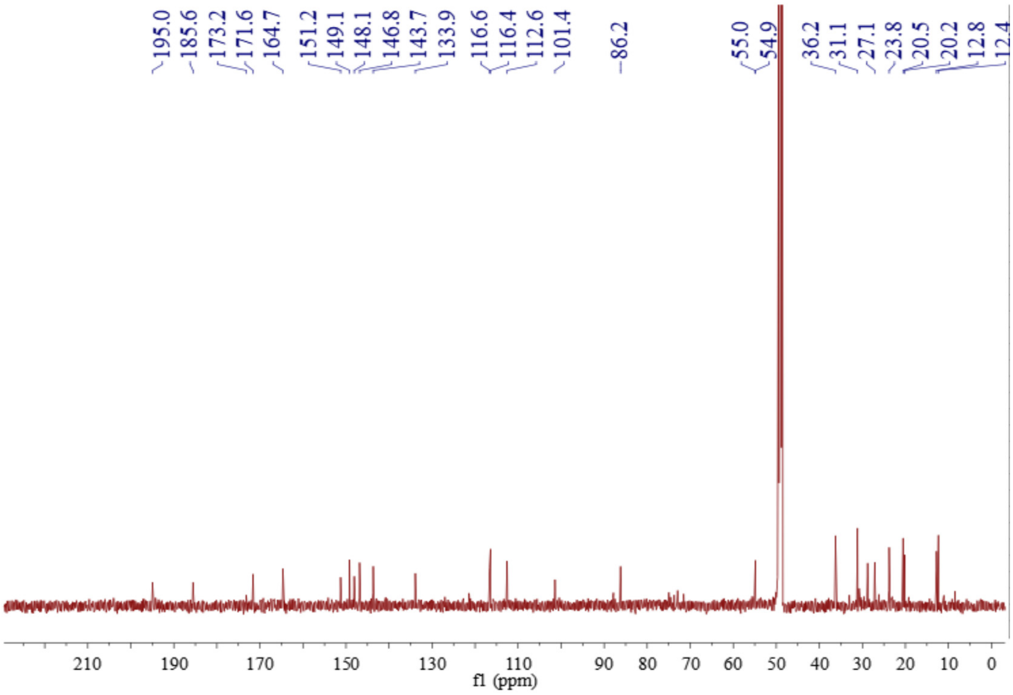

Figure S19. The DEPT135 spectrum of penazaphilone L (**3**)

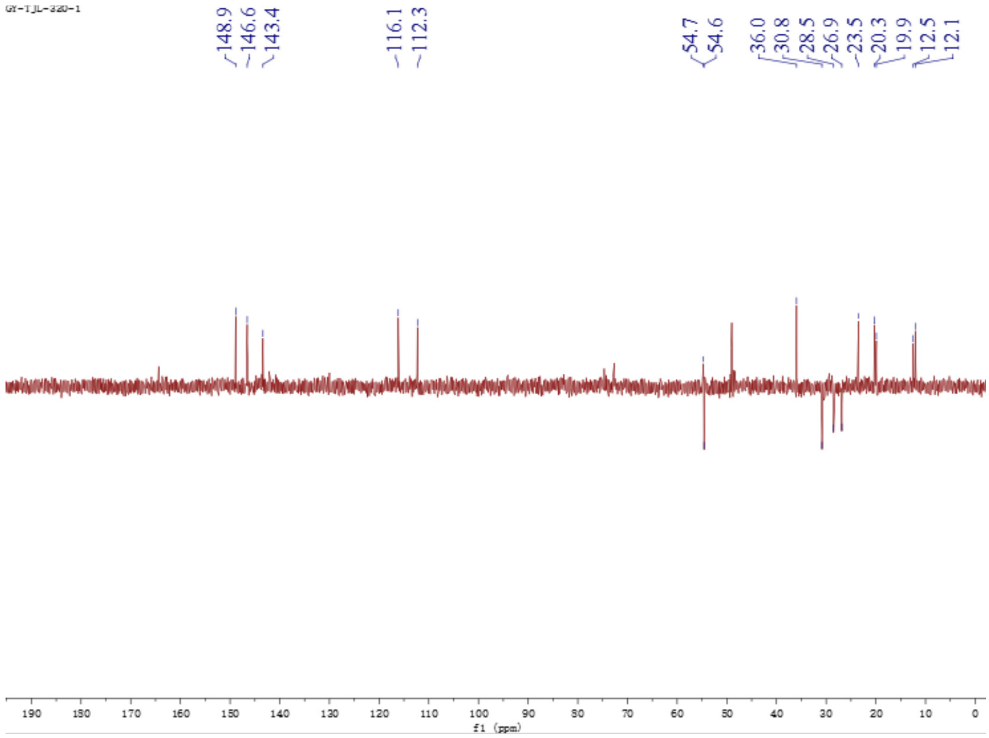

Figure S20. The HSQC spectrum of penazaphilone L (**3**)

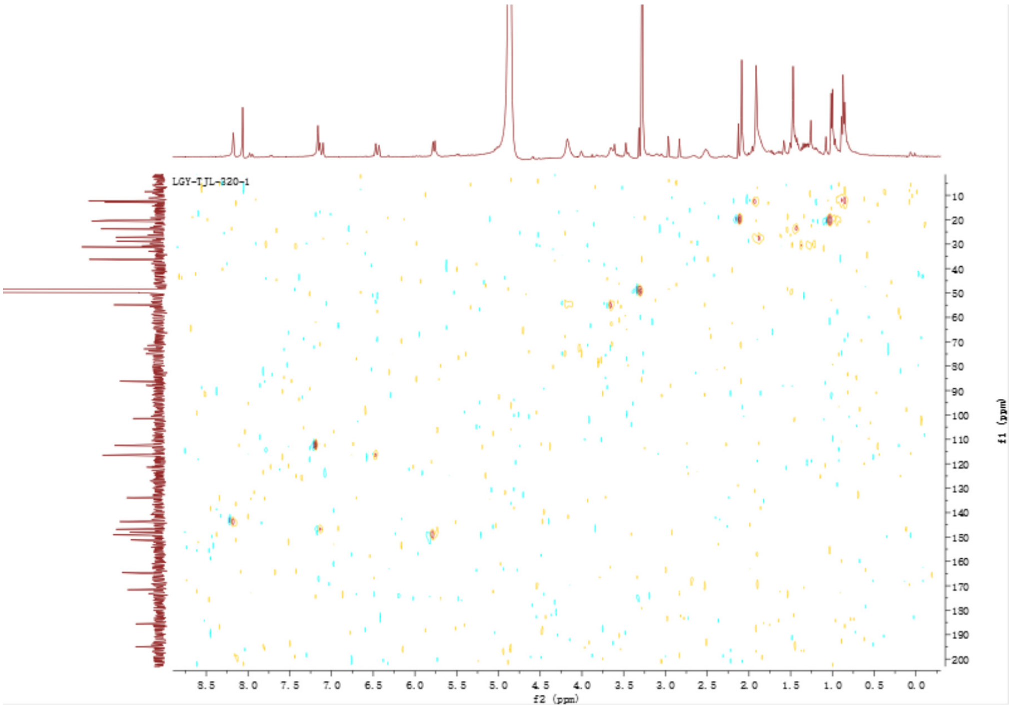

Figure S21. The HMBC spectrum of penazaphilone L (**3**)

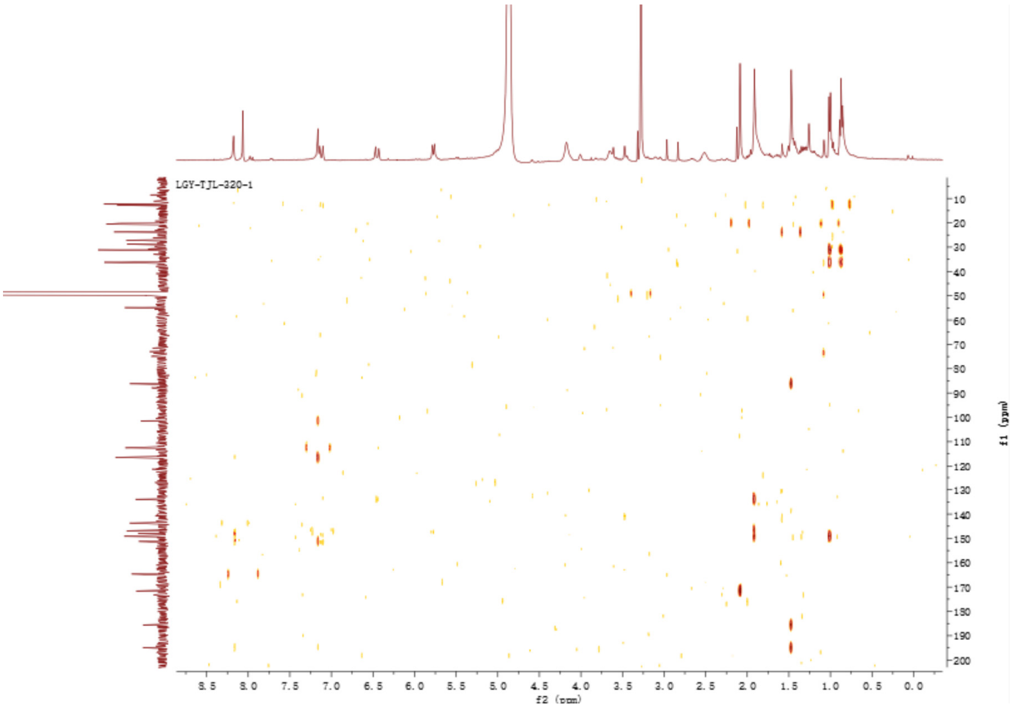

Figure S22. CD spectrum of penazaphilone L (**3**)

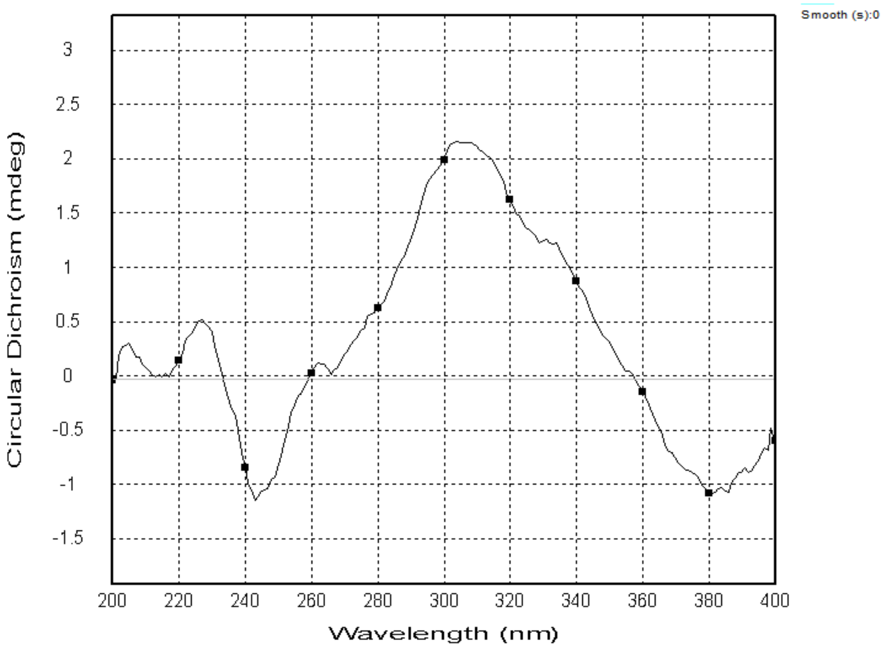

Table S1 The sequences of primers for qRT-PCR

Names	Sequences
GAPDH	5′-CACTCACGGCAAATTCAACGGCA-3′
	5′-GACTCCACGACATACTCAGCAC-3′
COX-2	5′-CACTACATCCTGACCCACTT-3′
	5′-ATGCTCCTGCTTGAGTATGT-3′
iNOS	5′-CCCTTCCGAAGTTTCTGGCAGCAG-3′
	5′-GGCTGTCAGAGCCTCGTGGCTTTGG-3′
IL-6	5' -AAGTGCATCATCGTTGTTCATACA-3'
	5'- GAGGATACCACTCCCAACAGACC-3'
IL-1β	5' -TGAAGCAGCTATGGCAACTG-3'.
	5'- AGGTCAAAGGTTTGGAAGGA-3'
